# Efficacy and safety of oral ivermectin in the treatment of mild to moderate Covid-19 patients: a multi-centre double-blind randomized controlled clinical trial

**DOI:** 10.1186/s12879-024-09563-y

**Published:** 2024-07-22

**Authors:** Ananda Wijewickrema, Hasini Banneheke, Arunasalam Pathmeswaran, Fathima Wardha Refai, Malika Kauranaratne, Neelika Malavige, Chandima Jeewandara, Mahendra Ekanayake, Dilhar Samaraweera, Dhanusha Thambavita, Priyadarshani Galappatthy

**Affiliations:** 1 National Institute of Infectious Diseases, Angoda, Sri Lanka; 2 Department of Parasitology, Faculty of Medical Sciences, University of Sri Jayewardenepura, Nugegoda, Sri Lanka; 3 Department of Public Health, Faculty of Medicine, University of Kelaniya, Ragama, Sri Lanka; 4 Department of Parasitology, Medical Research Institute, Colombo, Sri Lanka; 5 Department of Immunology and Molecular Medicine, Faculty of Medical Sciences, University of Sri Jayewardenepura, Nugegoda, Sri Lanka; 6 Base hospital Homagama and District Hospital, Wethara, Sri Lanka; 7 Colombo South Teaching Hospital, Kalubowila, Sri Lanka; 8 Department of Pharmacology, Faculty of Medicine, University of Colombo, Colombo, Sri Lanka; 9 North Wales Medical School, Bangor University, Bangor, UK

**Keywords:** Ivermectin, Double-blind, Randomized placebo-controlled clinical trial, RT-PCR-confirmed Covid-19 infection, World Health Organization Clinical Progression Scale, Viral load, Serum ivermectin levels

## Abstract

**Background:**

Evidence on ivermectin as a treatment for Covid-19 is controversial. A Cochrane review concluded that the efficacy and safety of ivermectin is uncertain (evidence up to April 2022) and WHO recommended its use only in the setting of clinical trials. This study aimed to assess the efficacy and safety of oral ivermectin in hospitalized patients with mild to moderate Covid-19.

**Trial design and methods:**

A double-blind, randomized placebo-controlled clinical trial was conducted among RT-PCR-confirmed, adults, hospitalised within the first four days of symptoms. Patients received oral ivermectin 24 mg or placebo daily for five days. RT-PCR was repeated on days five and ten. Clinical progression was monitored using the World Health Organization Clinical Progression Scale. Serum ivermectin levels were measured on days three, five, and seven. The primary outcome was the difference in the viral load between day zero and ten in the two groups.

**Results:**

Out of 1699 patients screened, 249 underwent randomization and 127 received ivermectin, and 122 placebo. D10 median viral load for E gene (IQR) was 2,000 copies/mL (100 − 20,500) with ivermectin (*n* = 80) and 4,100 copies/mL (1,000–65,600) with placebo (*n* = 81, *p* = 0.028), per protocol analysis. The difference in Log viral load between day zero and ten between ivermectin and placebo was 3.72 and 2.97 respectively (*p* = 0.022). There was no significant difference in the WHO clinical progression scale or the adverse effects. Ivermectin blood levels taken before or with meals were not significantly different. Only 7 and 17 patients achieved blood levels above 160ng/ML and 100ng/ML respectively and they did not achieve a significantly lower viral load.

**Conclusion:**

Although ivermectin resulted in statistically significant lower viral load in patients with mild to moderate Covid-19, it had no significant effect on clinical symptoms.

**Trial registration number:**

SLCTR/2021/020, Sri Lanka Clinical Trials Registry. 19/07/2021.

**Supplementary Information:**

The online version contains supplementary material available at 10.1186/s12879-024-09563-y.

## Background

Ivermectin is an antiparasitic drug indicated for the treatment of intestinal helminths and filariasis in humans and animals [1]. During the Covid-19 pandemic this inexpensive, widely available drug listed in the World Health Organization (WHO) list of essential medicines was tested for repurposing, based on scientific evidence showing antiviral properties against RNA viruses [2]. In-vitro studies [3] and in vivo studies on animal models [4] reported antiviral action against SARS-CoV-2. However, adequate viral clearance has been shown only with high serum drug levels, and whether patients achieve sufficiently high levels for viral clearance was questioned [5].

When taken orally, the plasma concentration of ivermectin is proportional to the dose [6], and the time to reach maximum concentration (t_max_) and t_1/2_ are approximately 4 h and 18 h respectively [7]. Although the product information leaflet recommends ivermectin to be taken on an empty stomach, it also states, there is up to 2.5-fold increase in bioavailability when taken with high-fat meals or food which is also supported by a few other research studies [1, 7].

Initial repurposing was based on non-peer-review observational evidence [8]. Later there were many randomised controlled trials and several meta-analyses giving varying conclusions on efficacy (beneficial [9–12], inconclusive [12] or non-beneficial [13–18]. A Cochrane review concluded that they are uncertain about the efficacy and safety (evidence up to April 2022) [19] and WHO recommended its use only in the setting of clinical trials [20].

The objective of this study was to find out whether ivermectin given at a dose of 24 mg daily for 5 days reduces the viral load and clinical outcomes in patients with mild to moderate Covid 19 infection. We also measured the ivermectin blood levels to find out whether administration of ivermectin with meals achieved higher blood levels compared to when taken before meals and to identify any correlation of blood levels to viral clearance.

## Methods

### Trial design, locations and participants

A double-blind, randomized controlled clinical trial of ivermectin versus placebo was conducted simultaneously at four healthcare institutions in Colombo district; the National Institute of Infectious Diseases (NIID)-Angoda, Base Hospital-Homagama, District Hospital-Wetara, and intermediate care centre (ICC)-Kandawala which was affiliated to Colombo South Teaching hospital, Kalubowila, enrolling participants from 29/07/2021 to 17/03/2022. During this study period, delta (sub-lineages AY.28 and AY.104) was the dominant variant of SARS-CoV-2 from July to October 2021 [21] and then Omicron up to the early part of 2022 [22].

### Inclusion and exclusion criteria

Patients over 18 years of age, hospitalized within 4 days of onset (as per patient’s history recorded by the doctor at the admission unit) of mild to moderate symptoms of Covid-19 and confirmed by SARS-CoV-2 reverse transcriptase- polymerase chain reaction (RT-PCR), were enrolled. The severity of patients was categorised according to the World Health Organization (WHO) severity criteria (the latest version available at the time of conducting the trial) [23]. Patients with severe disease were excluded. Pregnant females, breast-feeding mothers, those with HIV co-infection, known persons with an allergy to ivermectin or anthelmintics, patients with critical stage such as those who require mechanical ventilation or anticipated impending need for mechanical ventilation, patients recruited to any other trials simultaneously, and patients who have already been started on ivermectin were excluded. 

### Interventions

Ivermectin 6 mg tablets (manufactured according to Good Manufacturing Practice (GMP) by Popular Pharmaceuticals Limited, West Panthapath, Dhaka 1207, Bangladesh) from one single batch was supplied by the local agent (ABC Pharma Services (Pvt) Ltd, Colombo, Sri Lanka) for the study. The placebo tablet containing all inactive excipients to match the ivermectin tablets was manufactured by the State Pharmaceutical Manufacturing Corporation (SPMC, Sri Lanka), to be identical in appearance, shape, weight, taste, colour, smell, and texture. Recruited patients were randomly allocated into two groups (ivermectin arm and placebo arm). The allocation ratio was one to one (1:1). Each participant received oral ivermectin 24 mg (400 µg per kilogram of body weight for an average 60 kg patient) or placebo daily for 05 days. For all the interventions (medication, data collection, and investigations) the date of intervention /enrolment was considered as day zero. Both ivermectin and placebo arms received standard care according to a circular issued by the Ministry of Health which included symptomatic treatment (as and when necessary) with age-appropriate doses of paracetamol, omeprazole, domperidone, and salbutamol nebulisation. Ivermectin arm had 45/127 (35%) and the placebo arm 49/122 (40%) patients who continued with their long-term medication with antihypertensives, anti-diabetic and lipid-lowering drugs.

### Outcomes

The main primary outcome measure was the difference in the viral load (calculated using the natural log Cycle threshold (Ct) value of RT- PCR test for SARS-CoV-2) between D10 and day zero in the two groups. The viral load was calculated using the Ct values as done in a previously reported study [24]. The viral load was calculated using the formula given in the protocol of the paper by Chen *at al* (2021) with a revision of the cut-off Ct value for our study [24]. The maximum Ct value in our study was 40; the maximum Ct value in the reference paper was 45. Therefore, the formula to calculate log base 10 viral load was revised as - (40 - Ct)/ log (base2)10.

Clinical progression of the patient was also measured using WHO Clinical Progression Scale measured on days three, six, ten, 14, 21, and 28 [25].

Secondary outcomes included RT-PCR on day five, improvement of the symptoms by day six and by day ten of intervention using the WHO clinical progression scale, and adverse effects noted in the two arms. Each symptom was scored as absent (score 0), mild (1), moderate (2), or severe (3) [24].

The outcome of viral load could be studied only in those who were discharged at Day 10. This was a change to trial outcomes after commencement.

### Sample size

For sample size calculation, retrospective data from a sample of 30 hospitalized patients were analyzed using their Cycle threshold (Ct) values for SARS-CoV-2 RNA on day one (D1) and day ten (D10). The difference in the natural log Ct values of D1 and D10 had a mean of 0.23 and standard deviation of 0.27. The sample size was calculated to observe at least a 50% difference in the viral load between treatment groups (from 0.23 to 0.34), assuming a standard deviation of 0.3. A sample size of 106 per arm was determined to have 80% power to detect this difference at a significance of 0.05. The final sample size was calculated to be 236 (118 per arm) with a dropout rate of 10%.

Data Safety Monitoring Board (DSMB) evaluated all adverse events after 50% of patients were recruited and granted approval to continue the trial. Termination of study was to be decided by DSMB, trial related regulatory authorities, who was to be notified of any major adverse event.

### Randomization and double blinding

For randomization computer-generated random numbers were created by the statistician in variable blocks of four, six, or eight. Ivermectin or placebo tablets were packed into identically labelled packages numbered from 1 to 400 according to the computer-generated random numbers. The list was kept sealed and locked until the completion of the trial. The label of each pack indicated only the randomization number and directions for taking the tablets. Patients were screened for eligibility and recruited by medically qualified pre-intern research assistants guided by the clinical investigators under whose care the patients were admitted. The clinical and other data and the randomisation number were entered into each patient’s case record form (CRF).The study was blinded to participants, investigators, data analysts, healthcare providers and outcome assessors.

### Approvals

Ethics approval was obtained from Ethics Review Committee of Faculty of Medicine, University of Colombo, Sri Lanka (EC-21-EM02). The trial was registered at the Sri Lanka Clinical Trials Registry [26] (SLCTR/2021/020) before patient recruitment. Good clinical practice guidelines for the conduct of clinical trials in Sri Lanka of the National Medicine Regulatory Authority (NMRA) were adhered [27]. The protocol was approved by the Clinical Trials Evaluation Committee (CTEC) of NMRA (CT/P38/15/2021). Ministry of Health, Sri Lanka also approved the trial (ETR/AC/M3/33/2021). Relevant heads of each institution where the study was conducted granted administrative approval. Informed written consent was obtained from all participants.

### Data collection procedure

The research assistants recorded participants’ presenting complaints, medical history, vaccination status, and routine physical examination findings on days zero, six, ten, 21, and 28 days. At the time of starting the study, all patients with Covid-19 were kept in the hospital until they were RT-PCR negative but later on, they were discharged once asymptomatic. Depending on the duration of the hospital stay, data after discharge was collected via telephone interviews.

Clinical progression was measured using WHO clinical progression scale on days three, six, ten, 14, 21, and 28. Progression of symptoms related to Covid-19 and adverse events related to treatment were recorded by both the participant and research team independently on days zero, three, six, ten, and 14. Each symptom was scored daily by the participant as absent (score 0), mild (1), moderate (2), or severe (3) as was used in a previously reported study [25]. Development of hypoxia (SpO_2_ < 94%) was assessed on routine monitoring.

A nasopharyngeal swab was collected on days zero, five, and ten, for RT-PCR to check viral load (Ct value) and detection of single nuclear polymorphism associated with variants of concern of SARS-CoV-2 RNA. A sample of blood was tested on days zero and ten to check for antibody status to the virus and the levels. RT-PCR and antibody levels were done at the Department of Immunology and Molecular Medicine, Faculty of Medical Sciences, University of Sri Jayewardenepura, Nugegoda.

To determine the serum drug level and Maximum concentration (CMax), blood was collected on days three and five, four hours after administering ivermectin/placebo, and on day seven (after 48 h of finishing the course) among patients who consented. Ivermectin was given one hour before meal to 50% of the consenting patients in the first half of the study and with meals to the other 50% of patients in the next half of the study to observe the maximum concentration achieved due to the effect of food. Higher drug levels of over 160ng/mL and over 100ng/m reported in previous studies [5, 28] were used to determine whether patients with higher blood levels achieved a greater reduction of viral load. Drug level analysis was carried out at the Department of Pharmacology, Faculty of Medicine, University of Colombo using Liquid Chromatography Mass Spectrometry with a (LC/MS/MS) methodology using moxidectin as internal standard. The analytical method was developed and validated according to European guidelines [29].

Patients were discharged according to the Ministry of Health guideline prevailing at the time of discharge (Ref. Circular no. DDG/LS/CV-GL/2020, Ministry of Health, Sri Lanka) and as decided by the responsible consultant. Due to the early discharge, RT-PCR sample collection on days five and ten could not be performed in some patients.

### Statistical analysis

Summary statistical analyses by treatment groups are presented as the median and interquartile range (IQR) or mean and standard deviation (SD) for numeric variables and counts and proportions for categorical variables. Outcomes and laboratory findings of the groups were compared using two-sample t-test, Wilcoxon rank sum test, chi-square test, or Fisher’s exact test depending on the type of outcome. ANCOVA model was used to compare the log10 viral load at day 10 between the treatment groups after adjusting for the initial viral load. A *p*-value < 0.05 was considered significant. All analyses were done using R version 4.1.

We performed prespecified subgroup analysis to see whether those who achieved higher blood levels had increased viral clearance and whether those who had medicines with meal achieved higher blood levels than those who had before meals.

No exploratory analysis was done after the study.

## Results

**Fig. 1:**
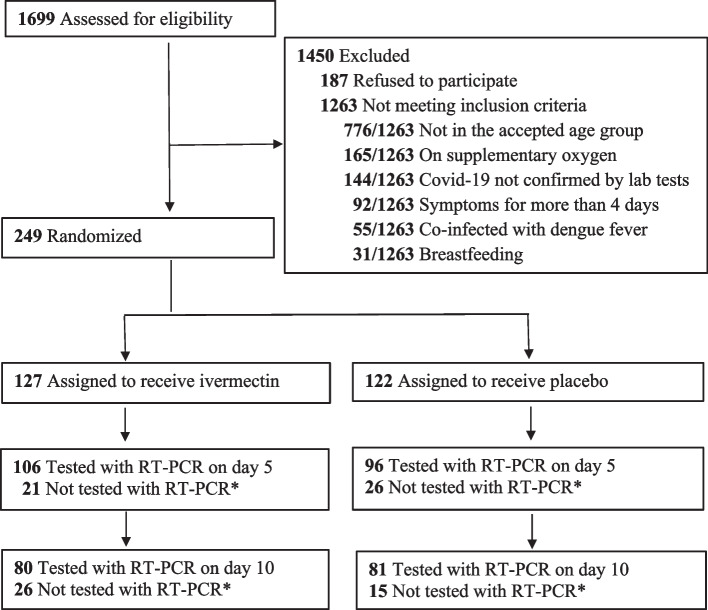
Consort diagram for the patients screened, randomized and completed during follow-up. *RT-PCR testing could not be carried out due to early discharge before day 10 and patients not returning for testing during lockdown and non-availability of swabs for a short period. (RT-PCR: Reverse-Transcription Polymerase chain reaction)

A total of 1699 patients admitted to study centers from 29/07/2021 to 28/03/2022 were screened for eligibility. Altogether 1466 were excluded (Fig. 1) and 249 were randomly assigned to receive ivermectin (*n* = 127) or placebo (*n* = 122). The median (interquartile range) hospital stay of the patients in ivermectin arms was 7 (5–8) days and the placebo arm was 7 (5–9) days. Baseline characteristics were similar in both groups (Table 1). Fever, headache, and myalgia were the commonest symptoms. Diabetes and hypertension were the commonest co-morbidities, each noted in about a quarter of the patients (Table 1). All participants continued their medication for the total duration.

**Table 1:** Baseline characteristics of the study population

Characteristic	Ivermectin *N* = 127 (%)	Placebo *N* = 122 (%)	*p*-value^a^
Age, mean (SD), years	42.4 (15.5)	46.3 (17.2)	0.073
Age 60 years or older (%)	20 (16)	32 (26)	0.039
Male	77 (61)	75 (61)	0.8
Vaccination status			
None	14 (11)	20 (16)	0.3
One dose only	7 (5)	8 (7)	
Two doses	105 (83)	89 (73)	
Missing	1 (1)	5 (4)	
Vaccine Type (2 doses)			
AstraZeneca	19 (18)	17 (19)	0.2
Sinopharm	79 (75)	65 (73)	
Pfizer	0 (0)	3 (3)	
Moderna	2 (2)	0 (0)	
Sputnik	4 (4)	1 (1)	
Not known	1 (1)	3 (3)	
Medical Conditions			
Hypertension	27 (21)	29 (24)	0.6
Diabetes	24 (19)	29 (24)	0.3
Dyslipidemia	4 (3)	11 (9)	0.052
Ischemic Heart Disease	4 (3)	9 (7)	0.13
Asthma	1 (1)	3 (3)	0.4
Symptoms on admission			
Fever	71 (56)	70 (57)	0.8
Headache	83 (65)	79 (65)	0.9
Myalgia	79 (62)	69 (57)	0.4
Runny nose	44 (35)	37 (30)	0.5
Sore throat	47 (37)	44 (36)	0.9
Dry Cough	65 (51)	57 (47)	0.5
Wet Cough	33 (26)	37 (30)	0.4
Difficulty in breathing	35 (28)	41 (34)	0.3
Abdominal pain	24 (19)	28 (23)	0.4
Diarrhoea	12 (9)	10 (8)	0.7
Nausea	20 (16)	8 (7)	0.022
Vomiting	8 (6)	2 (2)	0.10
Loss of smell	49 (39)	44 (36)	0.7
Loss of taste	46 (36)	49 (40)	0.5
Skin Rash	0 (0)	2 (2)	0.2

^a^Chi-square test except for age which was based on Welch Two Sample t-test

### Primary outcomes

The primary outcome on RT-PCR data was available in 80 patients in the ivermectin group and 81 in the placebo group for per protocol analysis. The mean Ct values were significantly higher, and the median log_10_ viral load and actual viral load was significantly lower in the ivermectin group compared to the placebo group at day 10 (*p* = 0.028) (Table 2).

**Table 2:** Virological endpoints on days zero, five and ten

Viral Load (E gene values)	Ivermectin	Placebo	Difference (95% CI^a^)	*P* value^b^
Day 0	*N* = 127	*N* = 122		
Ct value mean (SD)	18.3 (5.6)	17.9 (5.3)	0.4 (-0.9-1.8)	
Log_10_ viral load (copies/mL) mean (SD)	6.51 (1.67)	6.64 (1.59)	0.13 (-0.28-0.53,)	0.5
Viral load (copies/mL) median (IQR)	4,194,300 (262,100 − 33,554,400)	8,388,600 (1,048,600 − 67,108,900)		
Day 5	*N* = 106	*N* = 96		
Ct value mean (SD)	25.1 (6.5)	24.4 (6.4)	0.7 (-1.0-2.5)	
Log_10_ viral load (copies/mL) mean (SD)	4.47 (1.94)	4.70 (1.93)	0.23 (-0.31-0.76)	0.4
Viral load (copies mL) median (IQR)	65,500 (4,100–524,300)	65,500 (3,600–1,048,600)		
Day 10	*N* = 80	*N* = 81		
Ct value mean (SD)	30.1 (6.6)	27.7 (6.9)	2.4 (0.3–4.5)	
Log_10_ viral load (copies/mL) mean (SD)	2.98 (2.00)	3.69 (2.08)	0.71(0.08–1.30)	0.028
Viral load (copies/mL) median (IQR)	2,000 (100 − 20,500)	4,100 (1,000–65,600)		
Difference of log viral load between Day0 - Day10 (E gene)	*N* = 80 3.7 (2.0)	*N* = 81 3.0 (2.1)		0.022
Antibody status D0 Antibody status D10	68 / 120 (57%) 85 / 100 (85%)	62 / 116 (53%) 80 / 94 (85%)	0.75 (0.11, 1.4)	

^a^CI = Confidence Interval

^b^Welch Two Sample t-test

The difference in the log values of the viral load was equivalent to a five-fold greater reduction in the actual viral load in the ivermectin arm compared to the placebo arm.

**Fig. 2:**
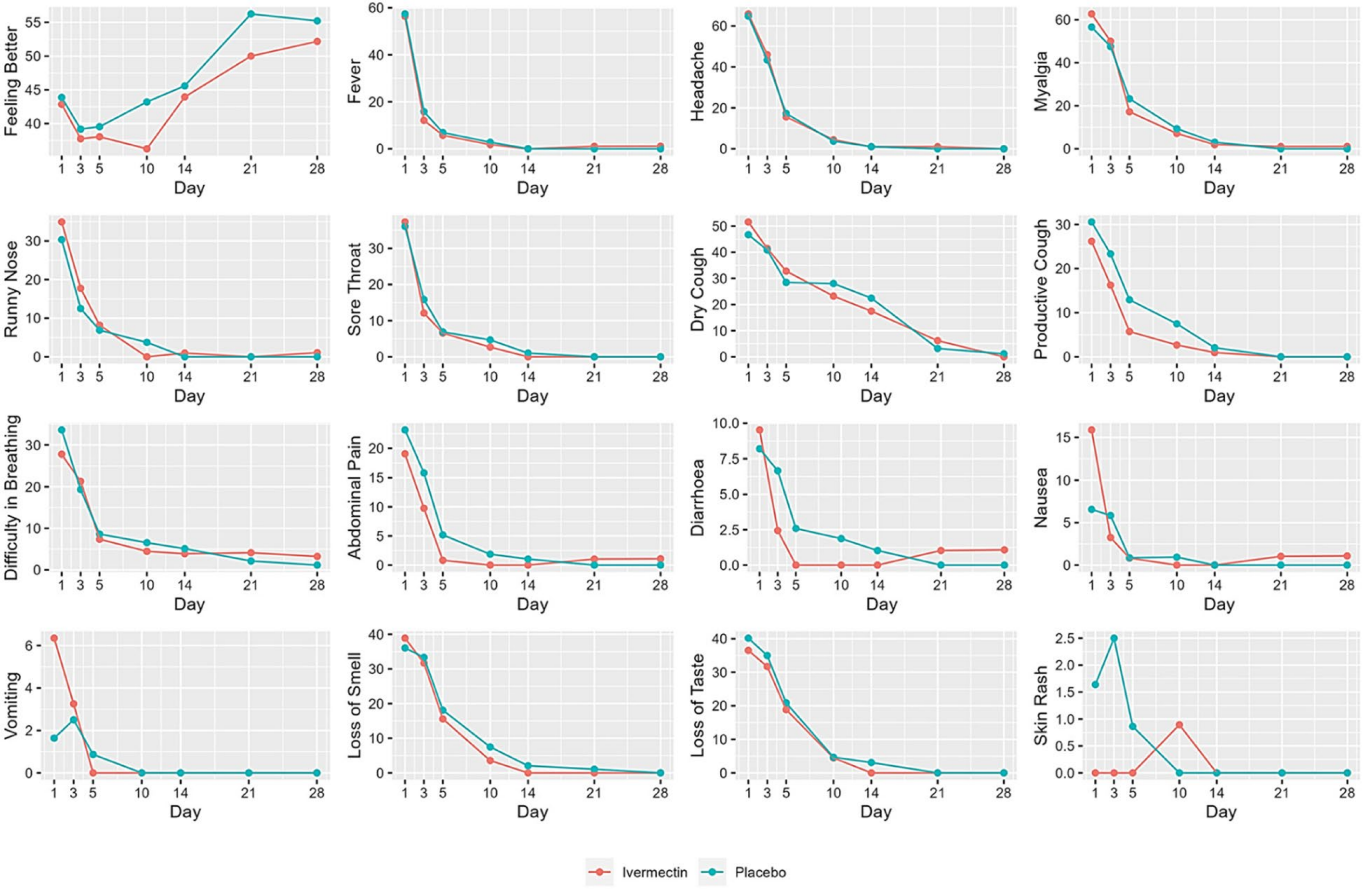
Progression of symptoms including possible adverse effects in the two groups (Ivermectin vs. placebo)

 Although viral load showed a significant reduction, there was no significant difference in the clinical progression of the disease according to the WHO clinical progression scale during the 28-day follow-up in the two arms (Table 3). Similarly, no difference was observed in the progression of clinical symptoms experienced by the patients during the follow-up period in the two groups (Fig. 2). These included specific symptoms such as loss of smell and taste as well as constitutional and respiratory symptoms, most of which were resolved by day 28. Nausea and vomiting were observed more in the ivermectin arm (Fig. 2) any serious adverse events were not noted with ivermectin. One patient died due to the development of severe Covid-19 pneumonia who was in the ivermectin arm, but it was not considered related to ivermectin.

**Table 3:** WHO clinical progression scale

Characteristic	Ivermectin, Number (%)	Placebo, Number (%)	*p*-value^a^
Day 0	*N* = 127	*N* = 122	0.8
2	121 (95)	112 / 120 (93)	
3	5 (4)	7 / 120 (6)	
Day 3	*N* = 120	*N* = 116	> 0.9
1	1 (1)	0 (0)	
2	29 (24)	27 (23)	
3 5	89 (75) 1(1)	89 (77)	
Day 5	*N* = 118	*N* = 113	> 0.9
1	3 (3)	4 (4)	
2	50 (42)	46 (41)	
3	64 (54)	63 (56)	
4	1 (1)	0 (0)	
Day 10	*N* = 106	*n* = 102	0.3
1	20 (19)	13 (13)	
2	58 (55)	54 (53)	
3	27 (25)	33 (32)	
4	0 (0)	2 (2.0)	
6	1 (1)	0/102 (0)	
Day 14	*N* = 99	*N* = 86	0.3
1	37 (37)	29 (34)	
2	46 (46)	46 (53)	
3	16 (16)	9 (10)	
4	0 (0)	2 (2)	
10	1(1)	0 (0)	
Day 21	*N* = 71	*N* = 60	0.4
1	49 (69)	43 (72)	
2	19 (27)	17 (28)	
3	3 (4)	0 (0)	
Day 28	*N* = 70	*N* = 59	0.4
1	55 (79)	49 (83)	
2	12 (17)	10 (17)	
3	3 (4)	0 (0)	

*N* number

^a^Pearson’s Chi-squared test; Fisher’s exact test

1. Mild disease asymptomatic; no limitation of activities

2. Mild disease Symptomatic; independent

3. Mild disease Symptomatic; assistance needed

4. Moderate disease but no oxygen therapy

6. Severe disease - on high-flow oxygen or non-invasive ventilation

10. Death

Although most patients had two doses of the Covid-19 vaccines only over half of patients had antibodies against Covid-19 at baseline. There was no difference in the antibody status between the two arms on day 10.

Ivermectin blood levels were available from most patients on Day 5 and there was no significant difference between the blood level achieved when the tablets were taken before meals or with meals (Table 4). There was wide variation noted in the blood levels achieved. Only a very small number of patients had higher blood levels of over 160ng/mL (*n* = 7) and over 100ng/mL (*n* = 17) and the viral load of those patients on Day 10 was not significantly lower compared to those who did not achieve these higher blood levels (*p* < 0.05) (Table 5).

**Table 4:** Ivermectin blood levels

Day (*n*)	Ivermectin blood level, Median (IQR), ng/ mL			*P* value^a^
	Total Sample	Ivermectin taken one hour before meal	Ivermectin taken with meal	
D3 (*n* = 31)	42 (25–55)	35 (5, 50) Range: (0 -314) (*n* = 10)	42 (31–58) Range (13–137) (*n* = 21) Range :13–137	0.3
D5 (*n* = 96)	25 (< 10, 60)	32 (< 10^b^, 79) *n* = 45 Range: 0-645	18 (< 10^b^, 41) *n* = 51 Range: 0-189	0.053
D7 (*n* = 28)	15 (< 10, 33) Range: 0-781	-	-	

^a^Wilcoxon rank sum test

^b^The level was below the lower limit of quantification of 10ng/mL

**Table 5:** Association of log10 viral load with the blood level of Ivermectin on selected days

Characteristic	High	Low	Difference	95% CI^a^	***p*-value**^**b**^
**Log10 Viral Load on Days 5 and 10 - by Ivermectin above 100 ng/mL (High)**					
D5 - log10 viral load E gene					
*N*	16	148			
Mean (SD)	4.8 (1.0)	4.6 (1.9)	0.28	-0.32, 0.87	0.4
(Missing)	1	13			
D10 - log10 viral load E gene					
*N*	13	121			
Mean (SD)	3.5 (1.3)	3.0 (2.0)	0.53	-0.34, 1.4	0.2
(Missing)	4	40			
**Log10 Viral Load on Days 5 and 10 – by Ivermectin above 160 ng/mL High)**					
D5 - log10 viral load E gene					
*N*	6	158			
Mean (SD)	5.2 (0.9)	4.6 (1.9)	0.60	-0.32, 1.5	0.2
(Missing)	1	13			
D10 - log10 viral load E gene					
*N*	5	129			
Mean (SD)	3.9 (1.9)	3.0 (2.0)	0.87	-1.5, 3.2	0.4

^a^CI = Confidence Interval

^b^Welch Two Sample t-test

## Discussion

This randomised double-blind controlled clinical trial of ivermectin 24 mg daily for 5 days compared to placebo, among patients with mild to moderate Covid-19, admitted to hospitals showed that there was a greater reduction in the viral load in the ivermectin group by Day 10 compared to placebo. However, there was no significant difference in clinical outcomes with ivermectin on the clinical progression and symptoms in the two groups, in keeping with most other recently reported well-conducted large-scale clinical trials [13–16]. This study provided data on certain important outcomes and aspects which were not subjected to investigation in initially reported trials. First, the primary outcome of this trial was viral load and at the time of designing this trial, evidence was sparse, although now more trials have reported on this outcome [5, 11, 28, 30–32]. Most of these studies showed a significant reduction in viral load or a trend in reduction, despite no significant effect on clinical outcomes. Our results are also in keeping with this same observation. Secondly, we studied the pharmacokinetics aspect of ivermectin to determine the serum ivermectin levels on D3, D5, and D7 and furthermore, whether giving ivermectin before meals or with meals achieved higher serum levels. Previous studies indicated that adequate viral clearance occurred only with high drug levels in serum [5]. Moreover, although the specification of product characteristics of ivermectin recommended administration one hour before meals, it also stated that high-fat meal increases blood levels, [1] which was supported by some studies [7]. However, our data did not show a significant difference in the serum levels in relation to meals. The meals consumed by most of our patients are rice (starch) based which is lower in fat content and this could have contributed to this observation. No correlation was also observed between high serum levels and viral clearance (Supplementary Table 2). Perhaps the small number of patients who achieved higher serum levels and the wide variation in the serum levels of ivermectin noted may have contributed to this observation. Ivermectin is highly protein bound and is metabolised in the liver undergoing oxidation in CYP 3A4 [1] and therefore the serum level is influenced by several drug-drug interactions with other concomitant medications, which could contribute to the very wide variation in serum levels observed. This trial also looked at patients’ antibody status at baseline and day 10. We noted that only about half of the patients had antibodies at baseline, despite approximately 80% receiving at least two doses of Covid-19 vaccines. Results also showed that over 80% of participants seroconverted by Day 10 (Table 2). We did not observe any difference in seroconversion rate with ivermectin.

There are many strengths in this study. We avoided the drawbacks reported in previous studies such as administration of the drug in the late stage of the illness, prescribing lower doses for too short a duration, inaccurate measurement of primary outcomes, and patients receiving other medications simultaneously with probable effects on SARS-CoV-2 virus. Further strengths include adherence to proper methodology including randomisation, double blinding, using RT-PCR based viral load as the primary outcome, superior statistical methods used for analysis, and measuring the ivermectin levels in the blood which had not been done in other studies.

We identified certain important limitations of the study. We could not perform Day 10 RT-PCR in 26 and 15 patients in ivermectin and placebo arms respectively. This was unavoidable as the discharge guidelines issued by the Ministry of Health changed during the study period to accommodate the increasing Covid-19 admissions during the pandemic and patients were discharged when clinical symptoms improved before Day 10. Some patients were unable to return for testing due to lockdown, restricted mobility, limited transport facilities, and financial constraints. Five patients in the ivermectin arm had day ten blood samples but RT-PCR tests could not be done due to the non-availability of RT-PCR sampling swabs in the country for a short period which was unavoidable. Furthermore, the sample size (106 in each arm) was calculated during the planning stage with the standard 80% power of the study. However, the data presented is of 80 (ivermectin) and 81 (placebo) patients. Table 2 presents the difference in Log_10_ viral load (copies/mL) on day 10; the post hoc power is 60%, which is lower than the planned conventional 80%, as we did not achieve the calculated sample size. However, this is unlikely to have affected the findings of this trial as we have observed a significant reduction in the viral load in the ivermectin arm. A proportion of patients did not have day 28 follow-up clinical information as they were not contactable over the phone after discharge. Since all patients were discharged home when they were clinically well, we assume that no adverse outcomes occurred following discharge. One patient in the placebo arm had taken hydroxychloroquine which was concluded to have no impact on the treatment of Covid-19 [33, 34]. Two in the placebo arm and one in the ivermectin arm had received dexamethasone prescribed by their general practitioner before admission. Systemic corticosteroids slightly reduce all-cause mortality up to 30 days in hospitalized patients with Covid-19 [35, 36].

## Conclusions

Ivermectin given at 24 mg daily for 5 days in patients with mild to moderate Covid-19, reduced the viral load significantly by Day 10 compared to placebo, but the clinical progression and symptoms did not differ in the two groups. The serum ivermectin levels varied widely but only a few patients achieved higher serum levels.

### Supplementary Information

Supplementary Material 1.

Supplementary Material 2.

## Data Availability

The datasets used and/or analysed during the current study are available from the corresponding author on reasonable request.

## Abbreviations

ADR: Adverse Drug Reactions
AE: Adverse Event
BNF: British National Formulary
CRF: Case Record Forms
Ct: Cycle threshold
D_0_: Day zero
DSMB: Data Safety Monitoring Board
ERC: Ethics Review Committee
FDA, USA: Food and Drug Administration, United States of America
FiO_2_: Fractional inspired Oxygen
GMP: Good Manufacturing Practices
MDA: Mass Drug Administration
MERS-CoV: Middle East Respiratory Syndrome Corona Virus
mg: milligram
mL: milliliter
NIID: National Institute of Infectious Diseases (IDH-Infectious Diseases Hospital)
NMRA: National Medicine Regulatory Authority
NPS: Nasopharyngeal swab
PaO_2_: Arterial Oxygen partial pressure
RA: Research Assistant
RCT: Randomized Clinical Trial
RT-PCR: Reverse Transcription Polymerase Chain Reaction
SAE: Suspected Adverse Event
SARS-CoV-2: Severe Acute Respiratory Syndrome Corona Virus 2
SEA: Serious Adverse Event
SLCTR: Sri Lanka Clinical Trail Registry
SpO_2_: Oxygen saturation
SPSS: Statistical Package for the Social Sciences (Statistical software package)
SUSAR: Suspected Unexpected Serious Adverse Reaction
USAR: Unexpected and Serious Adverse Reaction
WHO: World Health Organization
