# Supplementary material for: Efficacy and safety of oral ivermectin in the treatment of mild to moderate Covid-19 patients: a multi-centre double-blind randomized controlled clinical trial
Source: BMC Infect Dis. 2024 Jul 22;24:719. doi: 10.1186/s12879-024-09563-y (PMC11264372; doi:10.1186/s12879-024-09563-y)
Supplement: Supplementary file 1 — Supplementary Material 1. [file 12879_2024_9563_MOESM1_ESM.docx]

**Online-only Supplements**

**Supplementary Tables**

**Supplementary Table 1: WHO clinical progression scale**

| **Characteristic** | **Ivermectin, Number (%)** | **Placebo, Number (%)** | **p-value^a^** |
| --- | --- | --- | --- |
| Day 0 | N=127 | N=122 | 0.8 |
| 2 | 121 (95) | 112 / 120 (93) |  |
| 3 | 5 (4) | 7 / 120 (6) |  |
| Day 3 | N=120 | N=116 | >0.9 |
| 1 | 1 (1) | 0 (0) |  |
| 2 | 29 (24) | 27 (23) |  |
| 3  5 | 89 (75)  1(1) | 89 (77) |  |
| Day 5 | N=118 | N=113 | >0.9 |
| 1 | 3 (3) | 4 (4) |  |
| 2 | 50 (42) | 46 (41) |  |
| 3 | 64 (54) | 63 (56) |  |
| 4 | 1 (1) | 0 (0) |  |
| Day 10 | N=106 | n=102 | 0.3 |
| 1 | 20 (19) | 13 (13) |  |
| 2 | 58 (55) | 54 (53) |  |
| 3 | 27 (25) | 33 (32) |  |
| 4 | 0 (0) | 2 (2.0) |  |
| 6 | 1 (1) | 0/102 (0) |  |
| Day 14 | N=99 | N=86 | 0.3 |
| 1 | 37 (37) | 29 (34) |  |
| 2 | 46 (46) | 46 (53) |  |
| 3 | 16 (16) | 9 (10) |  |
| 4 | 0 (0) | 2 (2) |  |
| 10 | 1(1) | 0 (0) |  |
| Day 21 | N=71 | N=60 | 0.4 |
| 1 | 49 (69) | 43 (72) |  |
| 2 | 19 (27) | 17 (28) |  |
| 3 | 3 (4) | 0 (0) |  |
| Day 28 | N=70 | N=59 | 0.4 |
| 1 | 55 (79) | 49 (83) |  |
| 2 | 12 (17) | 10 (17) |  |
| 3 | 3 (4) | 0 (0) |  |

N=number

^a^Pearson's Chi-squared test; Fisher's exact test

1. Mild disease asymptomatic; no limitation of activities

2. Mild disease Symptomatic; independent

3. Mild disease Symptomatic; assistance needed

4. Moderate disease but no oxygen therapy

6. Severe disease - on high-flow oxygen or non-invasive ventilation

10. Death
