# Supplementary material for: Efficacy and safety of oral ivermectin in the treatment of mild to moderate Covid-19 patients: a multi-centre double-blind randomized controlled clinical trial
Source: BMC Infect Dis. 2024 Jul 22;24:719. doi: 10.1186/s12879-024-09563-y (PMC11264372; doi:10.1186/s12879-024-09563-y)
Supplement: Supplementary file 2 — Supplementary Material 2. [file 12879_2024_9563_MOESM2_ESM.docx]

**SupplementaryTable 2: Log10 viral load on days zero, five, and ten and association with blood level over100ng/mL and over 160ng/mL on days three, five, and seven**

| **Characteristic** | **High, N = 17** | **Low, N = 161** | **Difference** | **95% CI^a^** | **p-value^b^** |
| --- | --- | --- | --- | --- | --- |
| D5 - log10 viral load E gene |  |  |  |  |  |
| N | 16.0 | 148.0 |  |  |  |
| Mean (SD) | 4.8 (1.0) | 4.6 (1.9) | 0.28 | -0.32, 0.87 | 0.4 |
| (Missing) | 1 | 13 |  |  |  |
| D10 - viral load E gene |  |  |  |  |  |
| N log10 | 13.0 | 121.0 |  |  |  |
| Mean (SD) | 3.5 (1.3) | 3.0 (2.0) | 0.53 | -0.34, 1.4 | 0.2 |
| (Missing) | 4 | 40 |  |  |  |
| **Log10 Viral Load on Days 0, 5, and 10 - by over160 ng/mL** | | | | | |
| D5 - log10 viral load E gene |  |  |  |  |  |
| N | 6.0 | 158.0 |  |  |  |
| Mean (SD) | 5.2 (0.9) | 4.6 (1.9) | 0.60 | -0.32, 1.5 | 0.2 |
| (Missing) | 1 | 13 |  |  |  |
| D10 - log10 viral load E gene |  |  |  |  |  |
| N | 5.0 | 129.0 |  |  |  |
| Mean (SD) | 3.9 (1.9) | 3.0 (2.0) | 0.87 | -1.5, 3.2 | 0.4 |

^a^CI = Confidence Interval

^b^Welch Two Sample t-test
